# Topical Dexmedetomidine Pharyngeal Spraying Mitigates Severe Postoperative Sore Throat Following Rigid Bronchoscopy Despite Vocal Cord Injury: A Case Report and Clinical Insight

**DOI:** 10.7759/cureus.89989

**Published:** 2025-08-13

**Authors:** Yuguang Yang, Jun Lu, Lulong Bo

**Affiliations:** 1 Department of Anesthesiology, Changhai Hospital, Naval Medical University, Shanghai, CHN

**Keywords:** airway management, anesthesia complications, dexmedetomidine, postoperative sore throat, rigid bronchoscopy, topical application

## Abstract

Severe postoperative sore throat (POST) following rigid bronchoscopy (RB) is a common complication, with incidence rates reaching 62% due to mucosal trauma from large-diameter scopes. Conventional preventive measures often prove inadequate. This case report demonstrates the efficacy of topical dexmedetomidine (DEX) pharyngeal spraying in a 36-year-old male with recurrent tracheal adenoid cystic carcinoma and refractory POST after three prior RBs. Under bronchoscopic guidance, 48 µg of DEX, prepared by diluting 0.5 mL of 100 µg/mL commercial DEX solution in 4.5 mL of sterile saline, was sprayed onto both vocal cords before RB insertion. The POST severity was assessed using a validated four-point scale (0 = no pain; 1 = minimal discomfort; 2 = moderate pain affecting swallowing; 3 = severe pain with voice changes). Despite intraoperative vocal cord injury, the patient showed remarkable POST reduction (scores: 0 at 0/2/8h; 1 at 24h; 0 at 48h) and improved sleep quality, with stable hemodynamics and no adverse effects. The patient's history served as an intrinsic control, with prior RBs consistently causing severe POST despite conventional therapies. This targeted approach achieved localized anti-inflammatory effects through DEX's cytokine suppression while avoiding systemic risks. The intervention's success despite vocal cord trauma highlights its clinical robustness. Compared to systemic DEX, topical delivery uses lower doses while maintaining efficacy, offering advantages for complex airway procedures. These findings suggest topical DEX spraying may provide a novel preventive strategy in RB, particularly for high-risk patients. Controlled trials are warranted to validate this approach.

## Introduction

Postoperative sore throat (POST) is a frequent but often underestimated complication after airway instrumentation, with reported incidence rates ranging from 14.5% to 62% among affected patients [1-3]. POST usually stems from mucosal trauma caused by airway procedures, triggering localized inflammation. This can significantly impair patient satisfaction and recovery [4,5]. Rigid bronchoscopy (RB) is associated with an exceptionally high risk for severe POST due to the scope's large diameter and rigidity, which can cause substantial mucosal irritation. Conventional preventive strategies for POST, including corticosteroids, nonsteroidal anti-inflammatory drugs (NSAIDs), and local anesthetics, often yield suboptimal results for RB-associated POST [6-8], leaving a significant clinical challenge, particularly in patients with recurrent procedures or known susceptibility.

Dexmedetomidine (DEX), a selective alpha-2 adrenergic agonist, possesses potent anti-inflammatory properties alongside its sedative and analgesic effects [9]. Systemic (intravenous) DEX has been shown to reduce POST after standard intubation [10,11]. However, its use can be complicated by bradycardia and hypotension, which may limit its application in hemodynamically unstable scenarios or complex airway procedures. Topical application of DEX offers the potential for high local efficacy at the injury site while minimizing systemic exposure and side effects. Despite the high risk of mucosal trauma during RB, the feasibility and effectiveness of direct vocal cord application of DEX for this procedure remain unexplored.

This case report addresses a critical clinical dilemma encountered in managing a patient with a well-documented history of severe POST after multiple prior RBs, who presented for another complex RB procedure. We detail a novel, targeted approach using topical DEX spraying directly onto the vocal cords under visualization, implemented to mitigate the anticipated severe POST.

## Case presentation

A 36-year-old male (165 cm, 48 kg, American Society of Anesthesiologists (ASA) II) with recurrent tracheal adenoid cystic carcinoma (diagnosed in 2018) was scheduled for RB under general anesthesia. The procedure aimed to remove a silicone Y-stent and place a new Y-shaped covered metal stent. His complex medical history included eight prior flexible bronchoscopies with interventions and three prior RBs (stent placement in June 2022, removal seven months later, and silicone stent placement in October 2023 for recurrent stenosis and tracheoesophageal fistula).

Notably, all prior RB procedures were consistently followed by severe POST. Despite treatment with budesonide nebulization and lidocaine, the patient experienced persistent pain rated 2-3 on the four-point scale throughout recovery periods (0 = no pain; 1 = mild discomfort; 2 = moderate pain affecting swallowing; 3 = severe pain with voice changes). This level of pain significantly impaired his sleep and overall well-being. He expressed profound apprehension about experiencing POST again [12].

Preoperative assessment yielded no significant abnormalities. Airway evaluation, performed in accordance with standard protocols, disclosed a Mallampati class II classification, thyromental distance of 7 cm (within normal limits of 6.5-7.5 cm), interincisor distance of 4 cm (adequate for laryngoscopy), and full cervical spine mobility without evidence of restriction. Additional assessments, including neck circumference (38 cm), sternomental distance (12 cm), and hyomental distance (4 cm), were all within established normal ranges. No anatomical variations or pathologies predisposing to difficult airway management were identified.

**Figure 1:**
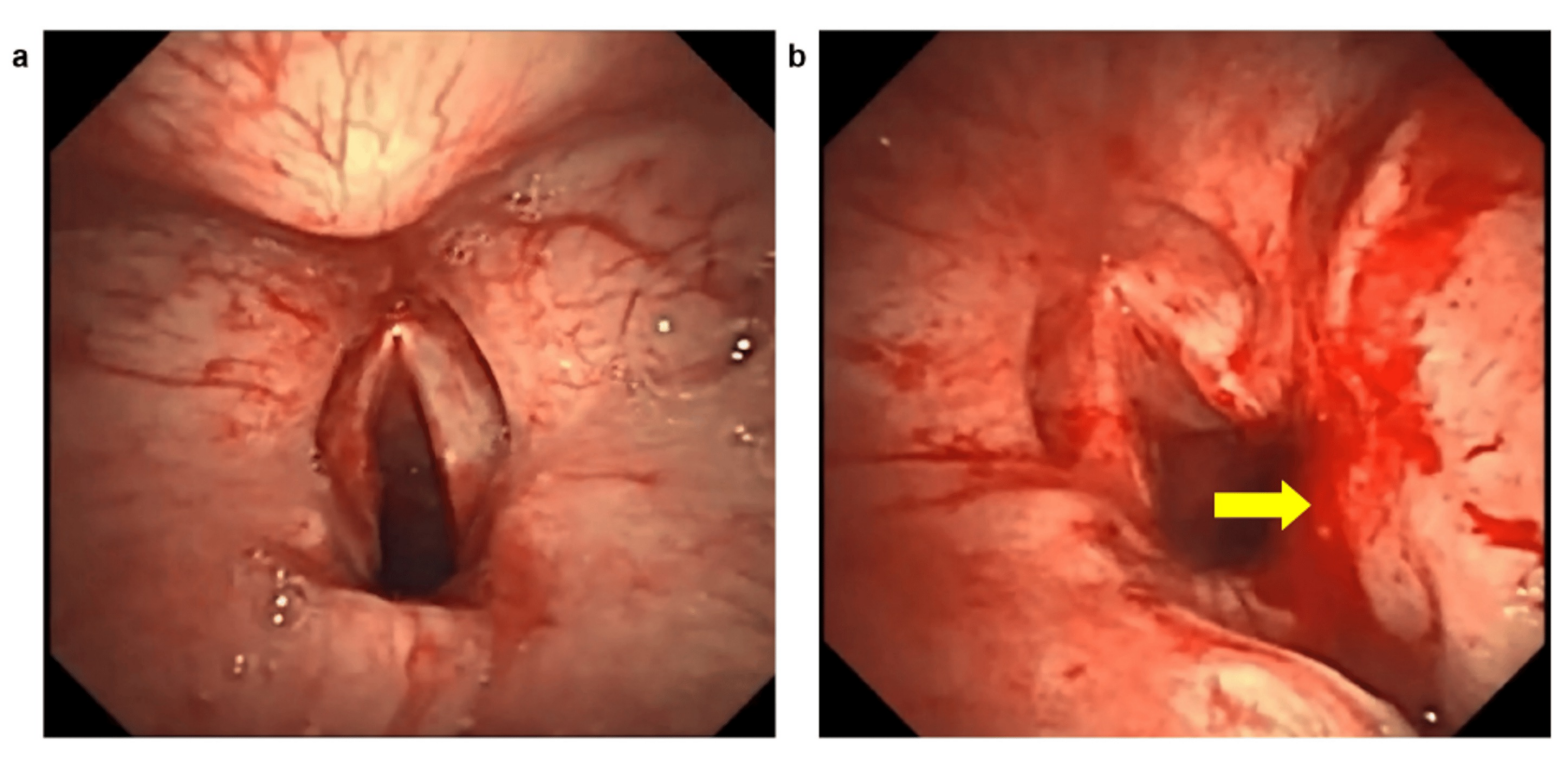
The glottis before and after the rigid bronchoscopy procedure. (a) The glottis before the procedure. (b) Post-procedure image showing a focal tear (yellow arrow) on the right vocal cord. Superficial epithelial tear (<2 mm). No submucosal hematoma or deeper injury noted.

Standard eight-hour fasting was implemented. Considering the patient's concern about postoperative sore throat, we did not administer anticholinergics to avoid potential dry mouth and other discomforts, aiming to maintain adequate mucosal hydration. Following preoxygenation, general anesthesia was induced with an intravenous administration of propofol (50 mg), fentanyl (0.2 mg), and rocuronium (45 mg), after which mask ventilation was initiated to maintain adequate oxygenation. Given the patient's high-risk profile for developing severe POST, a targeted novel intervention was implemented to mitigate this complication. Approximately three minutes after induction, following confirmation of optimal muscle relaxation, a flexible bronchoscope was inserted nasally, and under direct visualization of the laryngeal structures, a total of 48 µg dexmedetomidine (DEX; equivalent to 1 µg/kg) diluted in 5 ml normal saline was uniformly administered onto both vocal cords via the working channel of the bronchoscope, with the entire spraying procedure completed within 10 seconds. Representative image of the glottis prior to intervention is provided in Figure 1a.

The proceduralist then inserted the rigid bronchoscope (outer diameter 14 mm). During insertion, the rigid scope caused minor trauma (characterized as a "shoveling" effect) to the right vocal cord, resulting in a small tear and visible bleeding. This insertion was noted to be more challenging compared to previous instances. Anesthesia was maintained using target-controlled infusions of propofol (2-2.5 µg/ml) and remifentanil (5-5.5 ng/ml), with bispectral index (BIS) targeted between 40 and 60. No inhalational agents were used. Ventilation was facilitated via the side port of RB.

The procedure proceeded uneventfully thereafter: the existing silicone stent was extracted using rigid forceps, and a new metal stent was successfully deployed under fluoroscopic guidance to achieve fistula occlusion, with the entire procedure lasting 48 minutes.

Following RB removal, a size 4 laryngeal mask airway (LMA) (Well Lead Medical Co., Ltd., Guangzhou, China) was inserted. After confirming adequate ventilation, neuromuscular blockade was reversed (atropine 0.5 mg, neostigmine 2.5 mg). Spontaneous breathing returned within five minutes. Propofol was then discontinued. A flexible bronchoscope passed through the LMA confirmed the presence of vocal cord injury. A superficial mucosal tear (<2 mm) with punctate hemorrhage was identified on the mid-portion of the right vocal cord (Figure 1b). The patient regained responsiveness to verbal commands five minutes later. The LMA was then removed, and the patient was transferred to the post-anesthesia care unit (PACU). The interval from RB removal to LMA extraction was 12 minutes. Anesthetic depth was continuously monitored using BIS, with values maintained at 52 ± 3 (mean ± SD) throughout the procedure. Hemodynamics remained stable through the perioperative period, with the lowest HR at 78 bpm and the highest BP at 144/85 mmHg. No fluctuations >20% from baseline occurred.

The patient's recovery in the PACU proceeded smoothly without any adverse events. POST assessment by the four-point scale revealed a dramatic improvement compared to prior experiences: PACU arrival: 0; two hours: 0; eight hours: 0; 24 hours: 1 (mild dryness/soreness only on swallowing, no pain at rest); 48 hours: 0.

Notably, the patient explicitly reported a significant improvement in sleep quality during the first postoperative night, which he attributed to the absence of severe throat pain that had disrupted his rest following previous procedures. The patient remained clinically stable throughout the postoperative period and was transferred to the thoracic surgery service on postoperative day three to undergo the scheduled elective fistula repair procedure.​

## Discussion

This case report presents a novel solution to a recurrent and challenging clinical problem, severe POST following rigid bronchoscopy in a susceptible patient. The key clinical insight learned are as follows: (1) Topical application of DEX directly to the vocal cords immediately before RB insertion is feasible and safe in this setting; (2) This targeted approach can achieve a profound reduction in POST severity, even when an unforeseen complication-vocal cord injury-occurs during the procedure; (3) The localized delivery may minimize the hemodynamic side effects (bradycardia, hypotension) linked with systemic DEX administration.

The pathophysiology of POST involves mechanical trauma-induced inflammation. RB inherently poses a greater risk than standard intubation. DEX exerts potent anti-inflammatory effects, inhibiting pro-inflammatory cytokines and modulating immune responses [13]. DEX rapidly inhibits pro-inflammatory cytokines via α₂-adrenoceptors, offering mechanistic distinctions from corticosteroid genomic pathways. This may explain its efficacy in corticosteroid-refractory cases. Corticosteroids predominantly inhibit cytokine-mediated inflammation but show variable efficacy in POST after RB. This may arise from neurogenic mechanisms [14]: mucosal trauma activates sensory nerves, triggering neuropeptide release (e.g., substance P), thereby perpetuating pain hypersensitivity -- a pathway less responsive to corticosteroids. In contrast, dexmedetomidine's α₂-adrenergic receptor agonist action directly suppresses neurogenic inflammation by modulating neuronal excitability and neuropeptide release [13]. This distinction may explain the efficacy of DEX in corticosteroid-resistant cases. Systemic DEX reduces POST but carries hemodynamic risks. This case demonstrates that achieving high local concentrations at the site of potential trauma (vocal cords) via topical spray effectively suppressed the inflammatory response, as evidenced by minimal POST despite significant predisposing factors (RB procedure, known susceptibility, and actual vocal cord injury). Despite concerns about mucosal absorption, pharmacokinetic modeling based on Iirola et al. shows that DEX has 65% bioavailability after nasal mucosa administration [14]. The 48 μg dose results in systemic exposure of 31.2 μg (0.65 μg/kg for a 48 kg patient), below the hemodynamic threshold of 0.5-1 μg/kg. Even if absorption exceeds 65% due to mucosal trauma, the exposure (≤0.8 μg/kg) remains safe. This aligns with our observation of stable intraoperative vitals.

The patient's clinical history provided a compelling control; in prior RB procedures, severe POST consistently developed (with scores ranging from two to three) despite the implementation of conventional prophylactic and therapeutic measures, including nebulized corticosteroids and lidocaine administration [6,8,15]. The marked improvement (scores 0-1) with topical DEX, even in the presence of iatrogenic vocal cord injury, highlights the potential advantages of this targeted strategy over standard approaches for this specific challenge. A meta-analysis by Liu and colleagues corroborated the efficacy of systemic DEX administration in mitigating POST but concurrently highlighted its associated adverse hemodynamic risks, including a relative risk (RR) of 2.46 for bradycardia and 3.26 for hypotension [11]. Our case, in turn, provides preliminary evidence suggesting that topical DEX application may achieve comparable efficacy in preventing POST following RB while circumventing the systemic side effects inherent to intravenous administration.

## Conclusions

This case report offers a valuable clinical insight for managing challenging RB cases prone to severe POST: the topical spraying of DEX directly to the vocal cords under visualization prior to scope insertion appears to be a showed promising efficacy and safety preventive strategy in this case. Crucially, this approach demonstrated efficacy even when complicated by an intraoperative vocal cord injury, a scenario where systemic DEX might be problematic. This targeted intervention leverages DEX's potent anti-inflammatory properties precisely where needed, avoiding systemic side effects. However, this approach should be avoided in hemodynamically unstable or bradycardia-prone patients. These findings should be interpreted as preliminary insights; validation in larger cohorts is required before clinical adoption, particularly regarding optimal dosing.
